# Detection of Sleep Posture via Humidity Fluctuation Analysis in a Sensor-Embedded Pillow †

**DOI:** 10.3390/bioengineering12050480

**Published:** 2025-04-30

**Authors:** Won-Ho Jun, Youn-Sik Hong

**Affiliations:** Department of Computer Science and Engineering, Incheon National University, Incheon 22012, Republic of Korea; junwh_iot@naver.com

**Keywords:** humidity, sleep posture, rolling average, PELT algorithm, transfer learning

## Abstract

This study presents a novel method for detecting sleep posture changes—specifically tossing and turning—by monitoring variations in humidity using an array of humidity sensors embedded at regular intervals within a memory-foam pillow. Unlike previous approaches that rely primarily on temperature or pressure sensors, our method leverages the observation that humidity fluctuations are more pronounced during movement, enabling the more sensitive detection of posture changes. We demonstrate that dynamic patterns in humidity data correlate strongly with physical motion during sleep. To identify these transitions, we applied the Pruned Exact Linear Time (PELT) algorithm, which effectively segmented the time series based on abrupt changes in humidity. Furthermore, we converted humidity fluctuation curves into image representations and employed a transfer-learning-based model to classify sleep postures, achieving accurate recognition performance. Our findings highlight the potential of humidity sensing as a reliable modality for non-invasive sleep monitoring. In this study, we propose a novel method for detecting tossing and turning during sleep by analyzing changes in humidity captured by a linear array of sensors embedded in a memory foam pillow. Compared to temperature data, humidity data exhibited more significant fluctuations, which were leveraged to track head movement and infer sleep posture. We applied a rolling smoothing technique and quantified the cumulative deviation across sensors to identify posture transitions. Furthermore, the PELT algorithm was utilized for precise change-point detection. To classify sleep posture, we converted the humidity time series into images and implemented a transfer learning model using a Vision Transformer, achieving a classification accuracy of approximately 96%. Our results demonstrate the feasibility of a sleep posture analysis using only humidity data, offering a non-intrusive and effective approach for sleep monitoring.

## 1. Introduction

Sleep is a fundamental biological process, characterized by alternating cycles of non-rapid eye movement (NREM) and rapid eye movement (REM) sleep [1,2,3]. In typical sleep patterns, NREM sleep dominates the early phases of the night, while REM sleep increases toward morning, facilitating a natural wake-up cycle [4].

Previous research has identified body temperature and humidity as key environmental factors influencing sleep quality. The core body temperature ($T_{C}$), typically ranging from 36.5 to 37.5 °C, differs from the skin temperature ($T_{S}$), and both fluctuate across sleep stages [5,6,7,8]. Humidity affects evaporative cooling, disrupting the heat-transfer balance between $T_{C}$ and $T_{S}$, particularly within the thermoneutral zone [9]. High humidity levels are associated with reduced sleep efficiency and increased arousal, potentially by interfering with the thermoregulation and homeostatic processes involved in sleep induction and maintenance [10,11,12,13].

The optimal indoor humidity for sleep ranges between 30% and 50% [14]. Deviations from this range—especially high humidity—can disrupt REM sleep, impairing cognitive recovery and emotional regulation. Okamoto et al. [15,16,17] showed that exposure to humid heat during the first half of sleep significantly increases light sleep and awakenings while reducing slow-wave sleep (SWS), with diminishing effects later in the night.

Unobtrusive sleep monitoring systems have increasingly adopted sensor-based approaches. Haghi et al. [18] reviewed 21 studies using bed-based sensors, highlighting the heart rate and respiratory rate as the most commonly measured parameters. Chen et al. [19] and Ma et al. [20] introduced flexible humidity sensors capable of tracking breathing and motion during sleep, with applications in detecting conditions like sleep apnea.

Several studies have employed smart pillows with embedded sensors—typically pressure and temperature—to monitor sleep posture and movement [21]. Wearable devices such as the OURA ring have also been used to assess the influence of ambient humidity and temperature on sleep quality [22]. Other IoT-based systems monitor a broader range of bio-signals, including oxygen saturation, the heart rate, and snoring, to evaluate sleep health [23].

Several prior studies have explored smart pillow systems incorporating embedded sensors to monitor sleep-related parameters. For instance, Li et al. [24] extended earlier work [25] by integrating multiple temperature and humidity sensors—including zero-heat flux, dual-heat flux, LM35, and SHT20—into the pillow surface using latex-based supports. Their system also featured a 4 × 10 grid of 40 pressure sensors (FSR-406) for sleep posture recognition.

Veiga et al. [26] proposed a multi-modal sensing pillow that integrates temperature, humidity, luminosity, sound, and vibration sensors. Moshayedi et al. [27] presented a framework for evaluating pillows and mattresses tailored to individual users, employing heterogeneous sensors such as 12 thermal (DS18B20, Dallas Semiconductor, Dallas, TX, USA), 4 temperature-humidity (SHT10, OHMITE, Warrenville, IL, USA), and a single FSR sensor. Their system emphasized zoned sensor placement based on the transmission range, differing significantly from our approach, which focuses on uniform, linear sensor placement.

A related study by [28] investigated sensor placement on a smart cushion, emphasizing two-dimensional spatial optimization but without addressing spacing granularity—unlike our work, which considers the trade-off between the sensor count and posture resolution in a one-dimensional array.

Sensor placement impacts the system scalability and cost. Although the theoretical determination of the optimal sensor spacing is difficult, our method addresses this as a selection problem, balancing detail in posture detection with system complexity (see Section 4).

In terms of data processing, prior research has also emphasized sensor data normalization. Teh et al. [29] reviewed error-detection and correction techniques across sensor types, highlighting the importance of data consistency. Alemayou et al. [30] demonstrated that structured and normalized sensor data can improve feature extraction and classification performance in human-activity-recognition tasks.

This study aims to determine sleep postures in a non-intrusive and non-restrictive manner using humidity sensors embedded within a pillow. Building upon our previous work [31], which employed pressure sensors to monitor snoring and body temperature for distinguishing between REM and NREM sleep stages, this paper focuses on a novel approach: detecting tossing and turning during sleep solely through humidity data.

To explore the influence of humidity variations on sleep behavior, we developed a pillow embedded with a linear array of temperature and humidity sensors. These sensors enable the tracking of localized humidity changes throughout the night, offering insights into the sleeper’s environment, thermoregulation, and potential health indicators. Notably, the human body’s thermoregulatory efficiency decreases during REM sleep, often resulting in increased perspiration [32,33]. This localized increase in humidity can serve as an indirect marker for sleep stage transitions and physical movement.

Changes in body posture during sleep influence both heat distribution and moisture accumulation. Prior research has shown that airflow and thermal regulation significantly affect sleep quality by altering body temperature and perspiration levels [13]. Our observations revealed that humidity data exhibited greater temporal variation than temperature data, suggesting that it may serve as a more sensitive indicator of physical movement during sleep.

Recent advances in wearable and non-contact physiological monitoring have leveraged AI-integrated sensor systems to capture subtle biometrics in real time [34]. These approaches align with our aim to non-invasively detect sleep posture changes using humidity-based sensing.

In this study, we quantified fluctuations in humidity data using a rolling average approach that captures holistic sensor activity rather than isolated sensor events. To enhance detection accuracy, we employed the Pruned Exact Linear Time (PELT) algorithm to identify significant change points in the time series data.

Previously, our smart pillow system integrated eight pressure sensors (FSR-406, Interlink, Fremont, CA, USA) aligned in a linear configuration to infer sleep posture. While this paper builds on preliminary findings presented at a prior conference [35], it extends those concepts with a more detailed methodological framework and implementation strategy.

The central contribution of this work lies in demonstrating a strong correlation between aggregated changes in humidity sensor data over fixed intervals and alterations in sleep posture. This highlights the potential of using cumulative humidity variation as a reliable, non-invasive indicator for monitoring sleep dynamics.

## 2. A Pillow with Embedded Sensors and an Overall System Architecture

**Figure 1:**
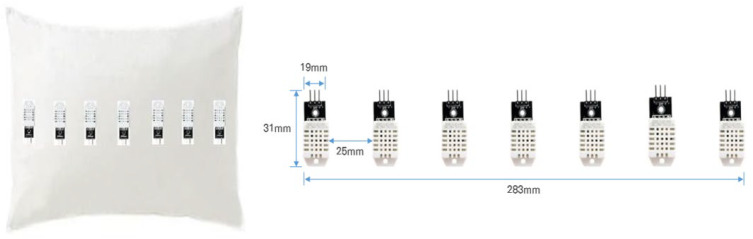
A pillow with built-in temperature and humidity sensors.

When the position of the head on a pillow moves during sleep, we aim to monitor changes in sleeping posture based on the resulting variations in humidity. Considering the head position on a pillow while lying down, a total of seven temperature and humidity sensors (DHT-22, Adafruit, New York, NY, USA) [36] were arranged in a row as shown in Figure 1.

Referring to Figure 1, it gives the sizes and intervals of the sensors arranged in the smart pillow. Based on Korean males aged 20 to 60, the head circumference ranges from 566 mm to 580.3 mm, a difference of 14.3 mm. Accordingly, we set the spacing between sensors to 25 mm. Since the spacing between sensors is difficult to prove theoretically, we approached it as a selection problem of optimizing the number of sensors discussed in Section 4.

We used a memory foam pillow that can absorb external shocks. The dimensions of the pillow are 420 mm × 220 mm. The temperature and humidity sensor model are AM2302 (DHT22), with a temperature measurement range of –40 °C to +80 °C (accuracy ±0.5 °C) and a humidity measurement range of 0% to 100% (±2%). Each sensor has the dimension of 25 mm × 40 mm, and the spacing between the sensors is 28 mm.

**Figure 2:**
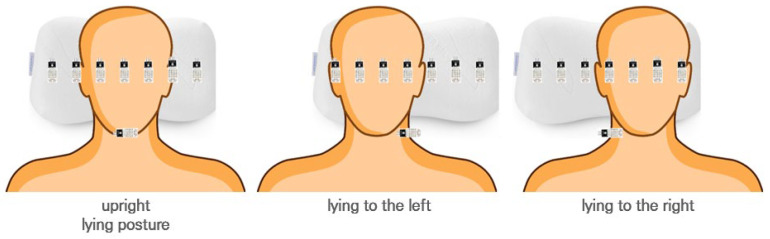
The three sleeping postures to classify using the pillow.

Changes in sleeping posture due to tossing and turning during sleep can be categorized into three types, as shown in Figure 2. The sleeping posture can be determined based on changes in humidity beneath the head. A temperature and humidity sensor can be used to monitor the subjects’ sleep quality.

**Figure 3:**
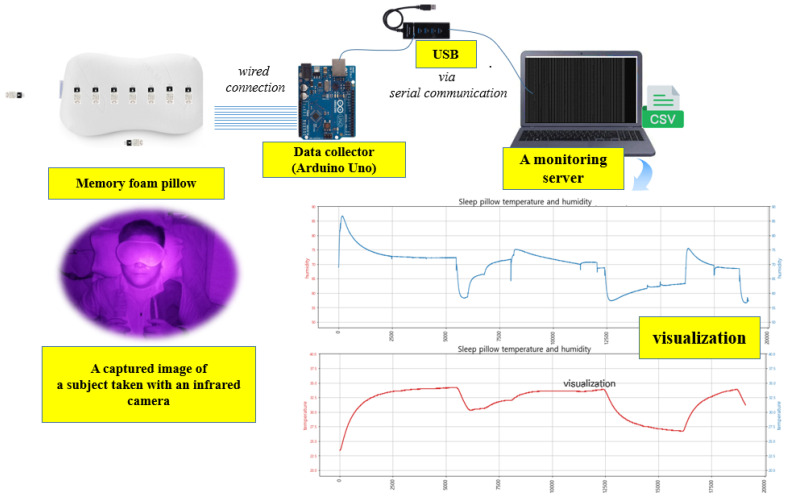
The overall system architecture.

The overall system configuration for acquiring raw data is shown in Figure 3. Two additional DHT22 sensors were placed around the subject to measure and compare not only the temperature and humidity where the head touches the pillow but also the surrounding environment. An Arduino Uno was used as a data collector to gather temperature and humidity data. To ensure the stable transmission of measurement data to the monitoring server, a wired USB connection was adopted instead of a wireless one. The measurement data are saved in CSV format on the server. For the purpose of verifying the posture discrimination results, images of the subjects were collected at regular intervals using an infrared camera installing on the ceiling.

## 3. Determining Sleeping Posture According to Humidity Changes

**Figure 4:**
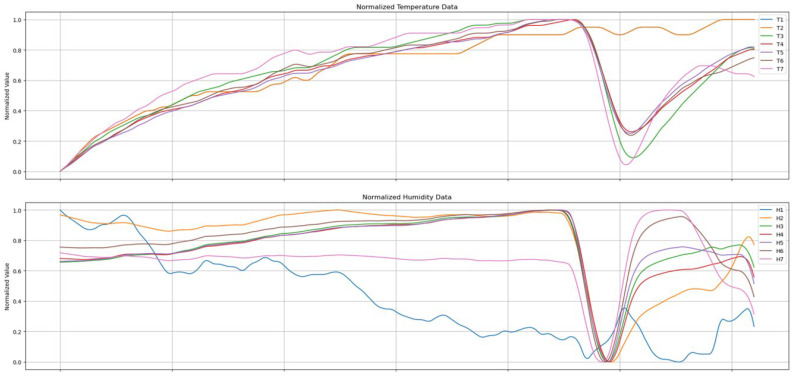
The temperature and humidity changes in the subject during sleep.

Changes in the temperature and humidity during sleep are shown in Figure 4. It presents the measurement results from the DHT22 sensor placed on the far left. As can be seen in Figure 4, while the temperature changes very slowly over time, the humidity changes relatively rapidly. We analyzed the characteristics of the temperature and humidity data and then conducted an experiment to determine whether the sleeping posture could be identified using only the humidity data. In particular, it can be observed that the humidity data measured by the seven sensors exhibited clear differences in their change curves depending on the sleeping position.

### 3.1. Comparison of Temperature and Humidity Changes

To quantitatively compare changes in temperature and humidity data over time during sleep, we calculated the average temperature and average humidity from the measurements of the seven temperature and humidity sensors. Based on these averages, we computed the absolute error (AE), which is the difference between the average value and each individual observed value. This deviation indicates how much each sensor’s measurement deviates from the average. The relative error (RE) is a measure of how much the data vary relative to the mean, expressed as a percentage as shown in Equation (1).

(1) $$Relative\;Error(\%)=\frac{absolute\;error}{mean}\times 100$$

**Figure 5:**
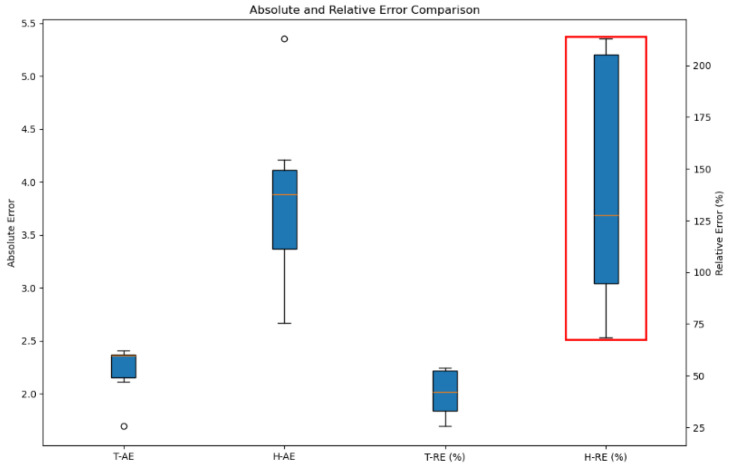
Absolute and relative errors of temperature and humidity data (The red box indicates the relative errors of the humidity data).

In Figure 5, T-AE and T-RE represent the absolute and relative errors of the temperature data, respectively. Similarly, H-AE and H-RE denote the absolute and relative errors of the humidity data. Since the humidity data exhibit a larger range of variation compared to the temperature data, it is evident that humidity changes can be sufficiently monitored even if the sampling interval is shortened. In other words, it is relatively easy to determine the sleeping posture based on changes in humidity.

### 3.2. Characteristics of Humidity Data by Location

**Figure 6:**
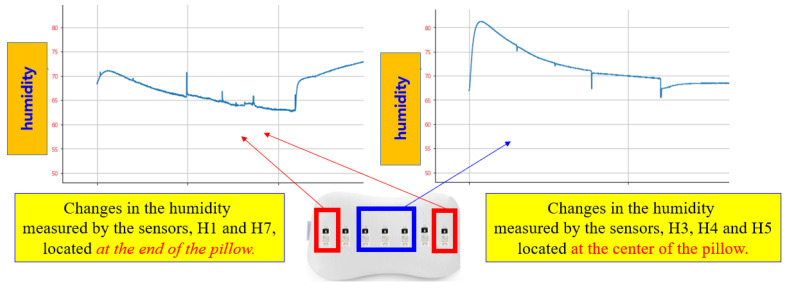
Changes in humidity data.

In Figure 6, the left curve shows changes in humidity data from the sensors 1 and 7, which are placed at both ends of the pillow. The right curve in Figure 6 displays the humidity data changes from the sensors H3, H4, and H5, located at the center of the pillow. The changes in humidity values of the sensors H1 and H7 differ from those of the sensors H3, H4, and H5. Therefore, it is evident that we can predict the sleeping posture based on changes in the humidity sensors when the head moves.

**Figure 7:**
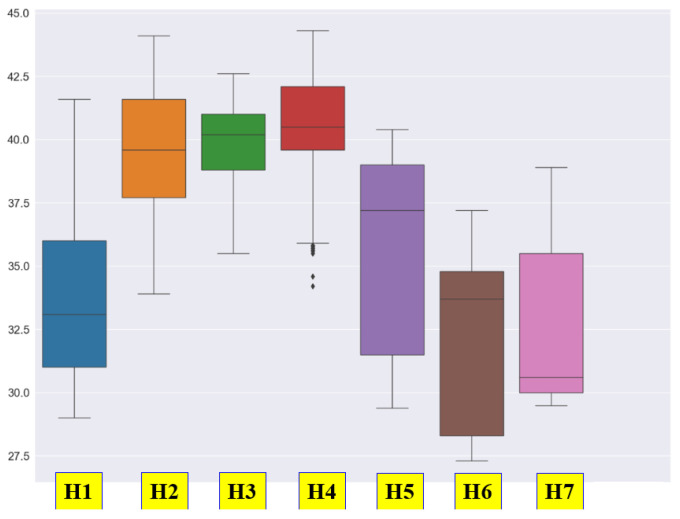
The box plot of the humidity data by location.

As shown in Figure 7, the box plot of the humidity data varies depending on the location of the sensors. Although there may be differences depending on the subject, it can be observed that the fluctuation range of the sensors placed at both ends is relatively larger compared to those of the sensors in the center.

**Figure 8:**
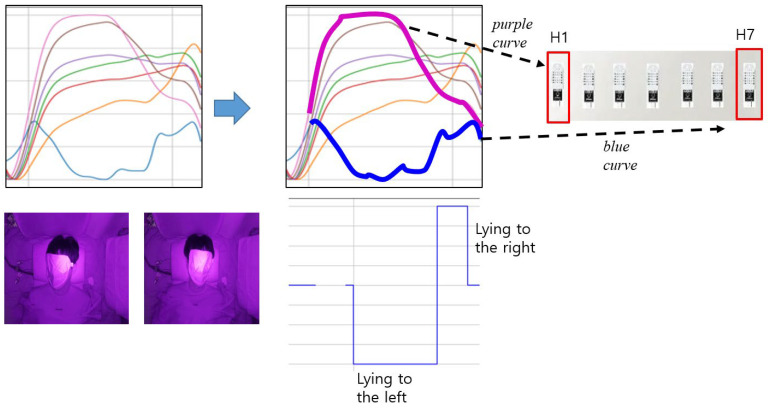
The change in humidity data for the sensor 1 and the sensor 7 over sleep time.

Humidity data change unevenly for each sensor at the point of change in sleeping posture as shown in Figure 8. In Figure 8, the purple and blue curves are the humidity change curves of H1 and H7, respectively. In addition, the reddish brown, dark purple, green, red, and orange curves are the humidity change curves of H2, H3, H4, H5, and H6, respectively. In particular, the humidity sensors, H1 and H7, placed at both ends of the pillow show different changes from other sensors. When lying to the left, the humidity of H1 increases, but the humidity of H7 decreases. Conversely, when lying to the right, the humidity of H1 decreases, but the humidity of H7 increases. In other words, it can be seen that the H1 and H7 change curves most sensitively reflect changes in posture.

### 3.3. Correlation Between Humidity Data Changes and Sleeping Posture

A rolling smoothing process, also known as a *moving average*, was applied to investigate the relationship between changes in sleep posture and cumulative changes using windows. It is a technique used in time series analysis to smooth out short-term fluctuations and highlight longer-term trends or cycles in data [37].

When the window size is ω, the smoothing equation can be expressed in Equation (2):

(2) $$S_{t}=\frac{1}{\omega }\sum_{i=t-\omega +1}^{t}x_{i}$$

Notice that $S_{t}$ and $x_{i}$ are the smoothed value at time t and raw data within a window, respectively. For this study, the window size of ω = 10 s was selected after testing various intervals. This configuration effectively reduced noise while retaining key features, such as the sudden humidity changes associated with posture transitions. Instead of focusing on the minute-by-minute fluctuations, the rolling smoothing process highlights the broader trend, such as whether humidity is generally rising or falling.

**Figure 9:**
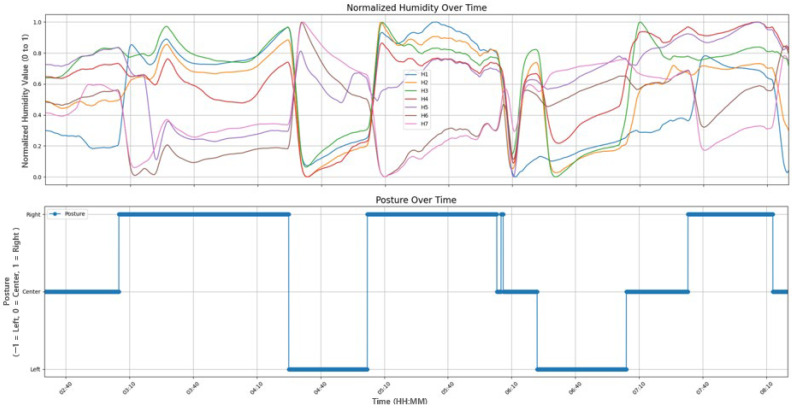
The relationship between the measured humidity data and the sleep postures.

At the top of Figure 9, the humidity data measured by seven sensors after preprocessing through rolling smoothing are displayed. Notice that the y-axis values in Figure 9 are normalized to range between 0 and 1. The bottom of Figure 9 shows a graph that determines the sleeping posture, with y-axis values representing right, supine, and left postures, respectively. As can be seen in Figure 9, a change in the sleeping posture occurred at the point when the humidity data changed

The humidity change patterns are largely driven by localized thermoregulatory perspiration and airflow disruptions caused by head movement. When the subject rotates the head to a different side, the new contact region absorbs moisture and restricts evaporation, leading to a rise in local humidity. Simultaneously, the previous contact region cools and releases moisture, resulting in a drop. This bidirectional change is most pronounced in the end sensors (H1 and H7), as shown in Figure 8. Exhalation effects were deemed negligible based on sensor positioning, and environmental confounds were controlled using ambient reference sensors.

### 3.4. The Analysis of Humidity Data Based on the Moving Average

We attempted to determine that the total amount of change in the seven-humidity data was closely related to the change in posture. We extracted the relationship between changes in humidity data measured by each sensor and changes in posture. When calculating the sum of changes in humidity data, we squared the differences to prevent them from canceling each other.

**Figure 10:**
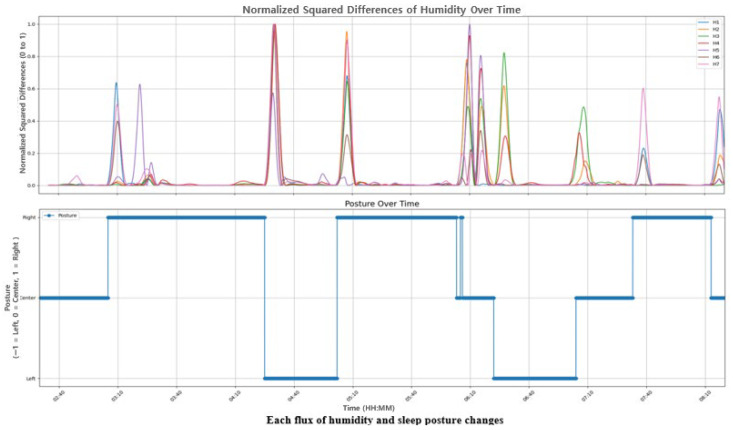
The relationship between the squared sum of changes in humidity data and the sleep postures.

The y-axis in Figure 10 represents the squared changes, normalized between 0 and 1. In other words, the y-axis value of 1 indicates the point of the maximum change of a sensor. Notice that the x-axis represents the sleep time. It displays all changes in humidity data measured by each of the seven sensors over time. As shown in Figure 10, a change in sleeping posture is indicated when the total amount of change exceeds a threshold (0.43).

This method involves calculating the average of a subset of data points (a “*window*”) and moving this window across the dataset to produce a new smoothed data series. We want to accumulate the standard deviations over a period from the time interval $\left\lfloor t\right.,\left.t+\Delta t\right\rfloor .$ It can be divided into n subintervals of width $\Delta t_{i}$ where i=1,2,…,n. For each subinterval, $\left\lfloor t_{i}\right.,\left.t_{i+1}\right\rfloor$, the standard deviation $\sigma_{i}$ can be calculated using the following Equation (3):

(3) $$\sigma_{i}=\sqrt{\frac{1}{N_{i}}\sum_{j=1}^{N_{i}}{\left(x_{j}-\mu_{i}\right)}^{2}}$$

where $N_{i}$ is the number of data points in the subinterval $\left\lfloor t_{i}\right.,\left.t_{i+1}\right\rfloor$, and $x_{j}$ are the individual data points within the subinterval. $\mu_{i}$ is the mean of the data in the subinterval, calculated as follows:

(4) $$\mu_{i}=\frac{1}{N_{i}}\sum_{j=1}^{N_{i}}x_{j}$$

Once we have the standard deviation for each subinterval $\sigma_{i}$, the accumulated sum of standard deviations $S_{\left\lfloor t_{i}\right.,\left.t_{i+1}\right\rfloor }^{\sigma }$ over the time interval $\left\lfloor t\right.,\left.t+\Delta t\right\rfloor$ for all the seven sensors is as follows:

(5) $$S_{\left\lfloor t_{i}\right.,\left.t_{i+1}\right\rfloor }^{\sigma }=\sum_{k=1}^{7}\sigma_{k}$$

We aim to consolidate the changes from the seven humidity data points into a single dataset to determine their relationship with sleeping posture. The *humidity flux* represents the total amount of change in data from all seven sensors. Humidity data are transmitted to the data collector every 10 s. In the moving average process, we use a window of 1 min, meaning that the average change is calculated over at least six data points and is reflected in the humidity flux.

**Figure 11:**
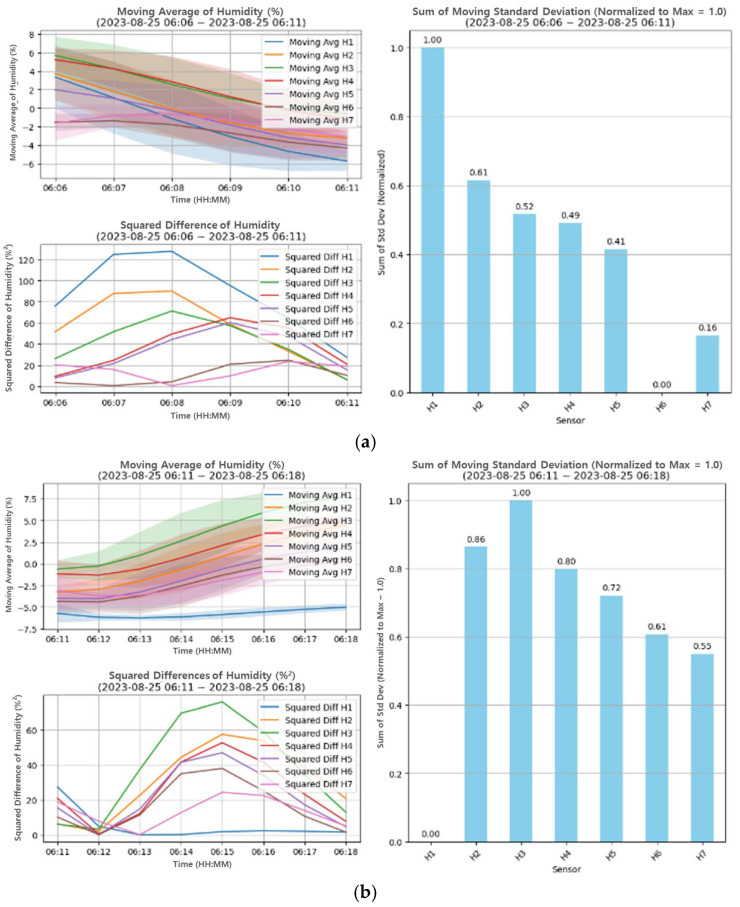
The change point detection applying the PELT algorithm; (**a**) the result of moving analysis sampled from 06:06 to 06:11; (**b**) the result of moving analysis sampled from 06:11 to 06:18.

Figure 11 shows the results of the moving average analysis for two consecutive intervals. Figure 11a,b show the results of moving analysis for two consecutive time intervals, which contains the moving average, the sum of squared differences, and the sum of standard deviations $\sigma_{i}$ of each sensor.

**Figure 12:**
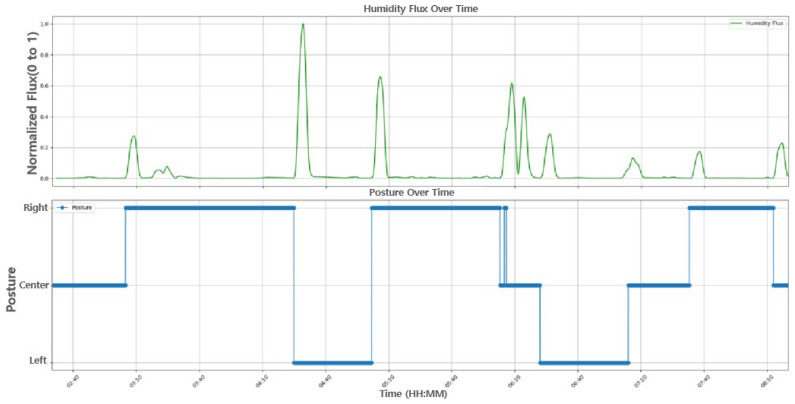
The relationship between the humidity flux and the sleep postures.

By applying a moving average to the humidity data, we derive its relationship with changes in sleeping posture, as depicted in Figure 12.

### 3.5. Applying the PELT Algorithm

We observed that posture changes occur in sections where the rolling average exceeds a certain threshold (0.16). Detecting these change points helps us understand the system’s behavior and make informed decisions. For this purpose, we will apply the PELT algorithm. The PELT algorithm is a powerful method used to detect multiple change points in a univariate time series [38].

It efficiently solves the optimal segmentation problem using dynamic programming combined with a pruning step to eliminate unnecessary computations. This approach guarantees finding the optimal set of change points that minimize the total cost function.

The $F\left(n\right)$ is the optimal total cost of segmentation up to time t and can be calculated as in Equation (6) [39]:

(6) Fn={ F τm+Cym+1, …, yn}τmmin

**Figure 13:**
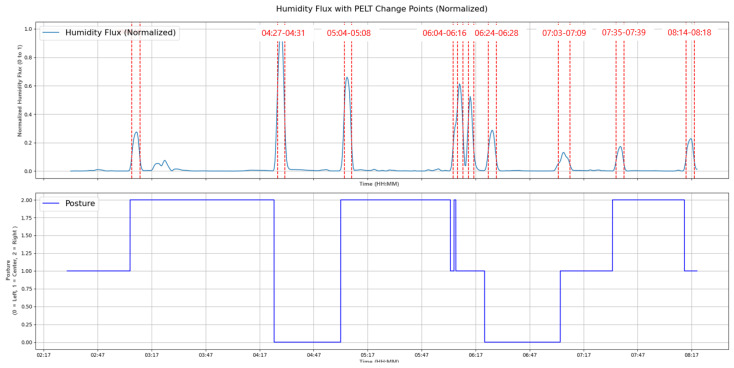
The change point detection applying the PELT algorithm for the subject with frequent postural changes.

Notice that $\tau_{m}$ denotes a candidate change point at position m. $F\left(\tau_{m}\right)$ is the optimal cost of segmentation up to the change point $\tau_{m}$. In addition, $C\left(y_{m+1},\ldots ,y_{n}\right)$ represents the cost associated with the segment of data from $y_{m+1}$ to $y_{n}$. It evaluates the data suitability of the interval and contributes to the overall cost to be minimized. In Figure 13, it can be seen that the PELT algorithm accurately extracts the posture change point section.

The PELT algorithm extracts posture changes from the accumulated change amount (flux) of a fixed window. When applying the PELT algorithm, the hyper parameter, *penalty,* can be obtained using a grid search method.

To determine the optimal penalty value for the PELT algorithm, we adopted a grid search strategy over a fixed interval (e.g., 0.05 to 0.5 with step size 0.01). The penalty parameter directly influences the number of detected change points, and improper tuning can lead to either over-segmentation or missed transitions.

Given the low dimensionality of the hyperparameter space (single scalar value) and the subject-specific variability in the tossing frequency, the grid search offers an interpretable and exhaustive method suitable for our application. This method ensures reliable posture transition detection even for subjects with subtle humidity variations. For each participant, the penalty value yielding the highest F1-score against ground-truth change points was selected.

**Figure 14:**
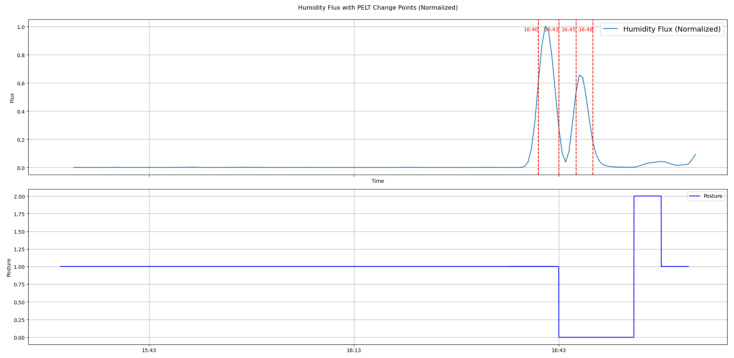
The change point detection applying the PELT algorithm for the subject with minimal postural changes.

The penalty varies depending on the subject. When the subject tosses and turns a lot during sleep as shown in Figure 13, the penalty can be set to 0.42 to determine posture changes. However, when the subject tosses and turns a little during sleep, as shown in the Figure 14, the penalty must be lowered to 0.112 to detect subtle changes.

To further validate the effectiveness of the PELT algorithm, we compared it with two representative change point detection methods: Binary Segmentation [40] and Bayesian Online Change Point Detection (BOCPD) [41]. All algorithms were applied to the same humidity flux time series data, and the resulting change points were compared against ground-truth posture transitions identified via infrared imaging. The evaluation was performed using a ±10 s tolerance window to account for minor temporal offsets.

As shown in Table 1, the PELT algorithm achieved the highest F1-score among all methods, indicating superior overall performance. Binary Segmentation, while computationally fast, tended to under-segment posture changes, missing subtle transitions. BOCPD exhibited moderate performance but was more sensitive to its prior settings and required careful tuning.

This comparative result justifies our choice of the PELT algorithm as the primary change-point-detection method in our study due to its robustness, accuracy, and consistent performance across different participants.

The penalty parameter in the PELT algorithm was determined per subject using a grid search approach, based on maximizing alignment with ground-truth transitions. While manual tuning may limit scalability, we propose integrating an automated estimation strategy using pre-sleep movement characteristics or real-time signal variability. Future work will also explore adaptive penalty formulations and heuristics to support real-world deployment across diverse users.

### 3.6. Experimental Conditions and Subject Information

Four individuals participated in the experiment, all of whom were male. The age distribution included three participants in their 20s and one in his 50s. We encountered difficulties in recruiting participants because the experiment required them to sleep for at least four hours. We obtained approval from the Institutional Review Board (IRB) for this study. All participants were fully informed about the experiment, and we received the consent forms that included their signatures.

### 3.7. The Humidity and Temperature of the Ears During Sleep

The state of sweating during sleep can be examined in relation to changes in sleep stages. However, we focused on humidity data and did not specifically investigate the relationship with sleep stages. Instead, we investigated whether changes in two sensors located at both ends of the humidity sensor were related to the ears.

The area around the ears can create a microenvironment due to limited air circulation, especially if the head is resting on a pillow [42]. Humidity around the ears might increase because the pillow traps heat and moisture. Skin near the ears may sweat minimally, contributing to local humidity without efficient evaporation.

Thermoregulation during sleep is closely linked to blood flow [43]. The body employs vasodilation and vasoconstriction to manage heat loss and retention. The ears, having a rich blood supply and being less insulated, may play a role in dissipating heat.

While specific studies on temperature and humidity within the external ear canal during sleep are scarce, it is recognized that the ear canal’s confined space can retain heat and moisture [44]. This retention may be influenced by ambient conditions, as discussed in research on thermal comfort in sleeping environments.

When sleeping on the side, air circulation and heat retention capabilities are reduced, so humidity may increase in the ear area pressed against the pillow. On the other hand, the other ear exposed to the air may have a lower relative humidity. This is consistent with the experimental results as shown in Figure 8.

## 4. Selection of an Optimal Number of Sensors

**Figure 15:**
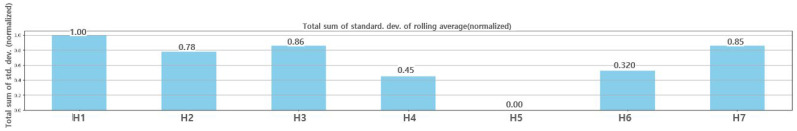
The normalized rolling averages for the sensors from H1(left) to H7(right).

Although seven sensors were placed at regular intervals, Figure 15 shows that there is a significant difference in the normalized rolling average for each sensor. When normalized to a maximum value of 1, the average is 0.63 with a standard deviation of 0.34, indicating a large variation in rolling averages between sensors. Interestingly, the rolling average increases as the sensor moves away from the center. Although these results may vary depending on the subject’s sleep patterns, it is necessary to check whether changes in the sleeping position can be monitored with fewer than seven sensors.

To determine the optimal sensor placement, we searched for sensor combinations that would produce results similar to using all seven sensors. In other words, when all the sensors were used, the PELT algorithm identified a total of 19 segments. We compared whether the same segments were found using sensor combinations ranging from a minimum of three sensors up to six. As summarized in Table 2, a total of ten sensor combinations were identified.

**Figure 16:**
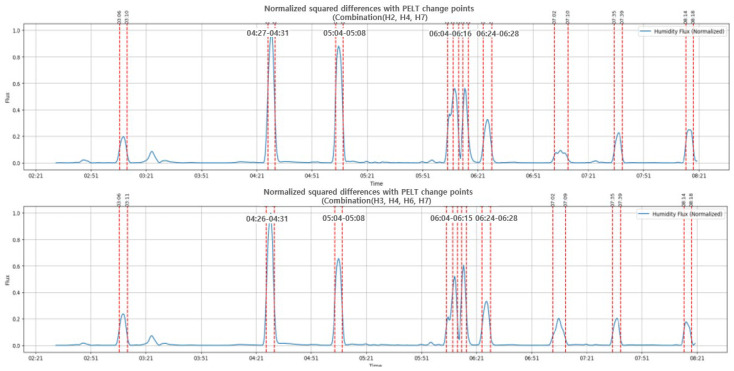
The results of applying the PELT algorithm to the set of three sensors (above) and the set of four sensors.

Figure 16 shows the results of applying the PELT algorithm when it is a combination of three sensors (H2, H4, H7) and a combination of four sensors (H3, H4, H6, H7).

While the sensor spacing of 25 mm was derived from head circumference data of Korean males, future designs will incorporate adjustable or flexible sensor layouts to accommodate variability in head sizes and pillow deformation across populations. A preliminary analysis using reduced sensor combinations also suggests that posture classification can be maintained with fewer sensors, indicating the spatial robustness of the system.

We created a dataset by converting the humidity data measured by each sensor into an image. A convolutional neural network (CNN) model was applied to this dataset to evaluate the performance of each sensor combination. The CNN model was custom-designed to identify the optimal sensor combination. By converting the humidity data curve over time into images, this CNN model was used to classify sleep positions. The CNN model consists of two convolution layers and max pooling layers. The first convolution layer uses 32 filters of size 3 × 3, while the second layer applies 64 filters to extract the main features of the image. These features are then learned through a flatten layer and a dense layer with 128 neurons, and final classification into the three positions is performed using the soft-max activation function.

**Figure 17:**
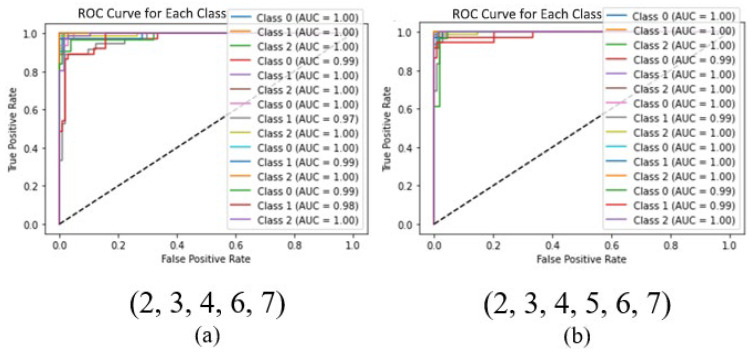
The ROC curves for the CNN models with the set of five sensors (**a**) and the set of six sensors (**b**).

Because data imbalance due to different postures negatively affected the model performance, weights were applied to reduce potential bias before evaluating the performance. There was no significant change in performance when using up to five sensors. However, with combinations of four sensors or fewer, the AUC value decreased significantly, indicating a decrease in performance. However, these results may be influenced by the subject’s sleep patterns. Figure 17 shows the ROC curves of the CNN models for two different sets of sensor combinations.

## 5. Experimental Results

**Figure 18:**
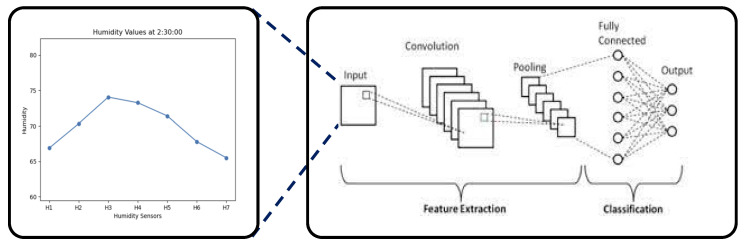
A sampled image from the humidity change curve.

For image classification, images that sampled the humidity change curve at specific intervals were extracted as shown in Figure 18. The dataset used to discriminate the sleeping posture consists of 2565 data points. The smart pillow collects humidity data every 10 s. A cycle for determining sleep posture can be arbitrarily set. In our experiments, sleeping postures are determined using accumulated humidity data over 10 min intervals.

A transfer learning model [45] that shows excellent performance in image classification tasks was applied. The Vision Transformer divides images into several patches, processes each patch like a token, and uses theses tokens as input to learn through the transformer architecture. To help the model better understand and process the images, each image was resized to 224 × 224 pixels. In the transfer learning process, to ensure that the model continues to maintain general characteristics, learning of the first 60 parameters of the model was disabled (these parameters were frozen). Additionally, the final classification layer predicts three classes: supine posture (lying on the back), right posture (lying on the right side), and left posture (lying on the left side).

**Figure 19:**
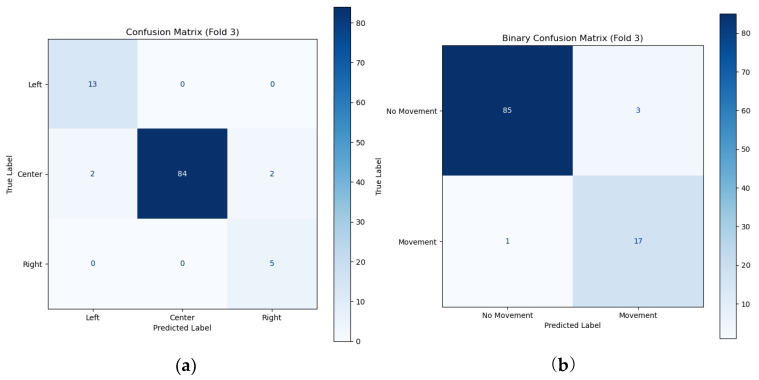
The multiple confusion matrix (**a**) and the binary confusion matrix (**b**) for the classification of sleeping postures.

The number of images used for learning is 528, of which 438 are images of sleeping in the supine posture, 66 are images of the left posture, and 24 are images of the right posture. The binary confusion matrix that determines whether a change in sleeping posture has occurred is as shown in Figure 19a. The evaluation parameters of the binary confusion matrix are an accuracy of 0.96, precision of 0.85, recall of 0.94, and F1-score of 0.89, respectively. The multiple confusion matrix that determines three sleeping postures as shown in Figure 19b gives a large difference in the discrimination accuracy of the left postures of 0.87 and the right posture of 0.71 compared to the precision of 1.00 of the supine posture.

**Figure 20:**
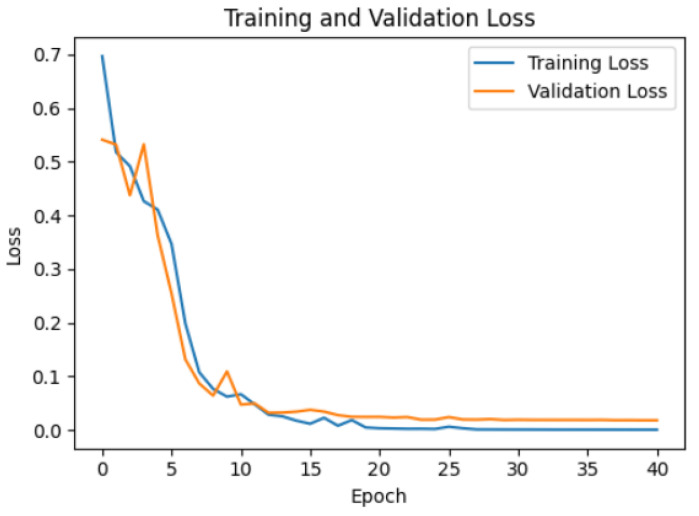
The training loss and validation loss for the model.

Eighty percent of the total dataset was used for training, and the remaining 20% was used for validation. A stochastic gradient descent optimizer was used, and a hyperparameter tuning process was performed. An accuracy of approximately 96.75% was achieved for the validation as shown in Figure 20.

## 6. Concluding Remarks

In this study, we proposed a novel method for detecting tossing and turning during sleep—an important factor in sleep pattern analysis—by leveraging an array of embedded humidity and temperature sensors within a memory-foam pillow. Our results showed that humidity data exhibit more pronounced temporal variation than temperature data, enabling the detection of head movement and posture changes based solely on humidity fluctuations.

We introduced a posture change detection algorithm based on the rolling sum of squared deviations across all sensors. A posture change was inferred when the smoothed signal exceeded a predefined threshold. To validate this approach, we employed the Pruned Exact Linear Time (PELT) algorithm to accurately identify change points within the time series data, confirming the strong correlation between humidity shifts and sleep posture transitions.

We further analyzed sensor effectiveness by evaluating various sensor combinations through PELT-based segmentation, enabling the design of customizable sensor configurations tailored to individual sleep profiles. While subject variability poses a challenge to generalization, our approach offers a framework for developing personalized smart pillows.

To enhance the classification accuracy, we converted time series humidity data into image representations and applied a transfer learning model for posture classification, achieving a mean accuracy of approximately 96%. This highlights the feasibility of using image-based deep learning approaches on physiological sensor data for sleep analysis.

Due to the highly imbalanced class distribution (438 supine, 66 left, 24 right), we applied a class-weighted loss function during training to reduce bias toward the majority class. The model was evaluated using stratified validation and a confusion matrix analysis, which demonstrated acceptable performance on minority classes. We froze the early layers in transfer learning to avoid overfitting, and future work will explore synthetic data augmentation to improve robustness.

Despite external factors such as the pillow material potentially influencing the sensor readings, our findings demonstrate that humidity sensing alone can serve as a reliable and non-invasive modality for detecting sleep posture changes. Our system can discern both the direction (left/right) and severity (mild/severe) of head movement during sleep. However, because detection is based on head motion, it currently lacks the capability to classify full-body postures or limb movements when head activity is minimal.

The limitation of the present study is that all participants were male, which may restrict the generalizability of the results. Future work will include a more gender-balanced cohort to explore whether biological differences influence humidity-based posture detection.

While the present study did not conduct a direct comparison with pressure or temperature sensors, our prior work and current observations suggest that humidity-based sensing offers higher sensitivity to subtle posture changes. Future research will incorporate simultaneous pressure–humidity–temperature measurements under controlled conditions to quantify their relative responsiveness and validate this hypothesis.

Future work will explore the integration of additional non-contact sensing modalities to extend detection capabilities beyond posture, including the identification of snoring and sleep apnea events, thereby contributing to more comprehensive sleep monitoring systems.

## Figures and Tables

**Table 1:** Change-point-detection algorithm comparison.

Algorithm	Precision	Recall	F1-Score
**PELT (Pruned Exact Linear Time)**	0.88	0.92	0.90
Binary Segmentation	0.74	0.83	0.78
Bayesian Online CPD (BOCPD)	0.79	0.81	0.80

**Table 2:** A set of sensor combinations matching the PELT results with seven sensors.

No of Sensors	No of Combinations	Sensor Combination Matching the PELT Results
3	2	(H2, H4, H7), (H3, H4, H7)
4	1	(H3, H4, H6, H7)
5	3	(H1, H3, H4, H5, H7), (H1, H3, H4, H6, H7), (H2, H3, H4, H6, H7)
6	4	(H1, H2, H3, H4, H5, H7), (H1, H2, H3, H5, H6, H7), (H1, H3, H4, H5, H6, H7), (H2, H3, H4, H5, H6, H7)

## Data Availability

All data used in this paper are dependent on the sensors used and the measurement environments. The measurement values of each sensor used in our experiments will be provided upon request via e-mail.
